# Conservative Management of a Rare Presentation of Mucosal Fenestration in a Four-Year-Old Child

**DOI:** 10.7759/cureus.47694

**Published:** 2023-10-25

**Authors:** R Veera Kumar, Nivethitha Karuppiah, Rajakumar A, Gayathri Gopinath, Govinda Rajaa

**Affiliations:** 1 Pediatric Dentistry, Priyadarshini Dental College and Hospital, Chennai, IND; 2 Oral Pathology and Microbiology, Priyadarshini Dental College and Hospital, Chennai, IND; 3 Rehabilitation Medicine, Government Institute of Rehabilitative Medicine, Chennai, IND

**Keywords:** periodontal inflammation, gingiva, fenestration, defect, alveolar bone

## Abstract

Mucosal fenestration refers to a window-like defect in the alveolar bone where the root of a tooth is denuded of its bony covering. Various causes ranging from trauma to chronic periapical or periodontal inflammation can produce such defects. This condition usually manifests either in adolescents or in extreme age group patients. The present case report is of a four-year-old boy who showed the presence of mucosal fenestration in the anterior maxillary gingivae in relation to teeth 51 and 61 (primary maxillary right and left central incisors as per the Fédération Dentaire Internationale (FDI) System) and was treated in a conservative and least invasive manner. Mucosal fenestration in the primary dentition phase can be treated in a conservative manner as opposed to any invasive treatment approaches, likely to instill fear or anxiety in a pediatric patient.

## Introduction

The term fenestration comes from the Latin word ‘fenestra,’ meaning window. Periodontal literature finds the usage of this term in describing areas in the alveolar process devoid of bone, creating a window revealing the underlying root surface [1]. The term gingival or mucosal fenestration is used in situations where the overlying gingiva or alveolar mucosa is denuded along with part of the alveolar bone exposing the root apex. Menendez, in 1967, described mucosal fenestration for the first time as bone fenestration by roots of deciduous teeth. Later, in 1976, Kelly et al. applied the term ‘apical fenestration’ [2]. According to Akbulut et al., the frequency of root fenestrations was 2% [3]. Mucosal fenestration can be attributed to various causes like tooth malposition, prominent morphology of root apex, thin or deficient alveolar bone, and severe chronic periapical inflammation leading to destruction of bone. The most common location of mucosal fenestration, as concluded from various studies, is the region of anterior teeth especially on the labial aspect of tooth angulation where the root apices are placed in a labial direction [4-6]. Usually, these cases are asymptomatic but may act as plaque-retentive areas, leading to inflammation of adjacent mucosa [7]. The diagnosis of apical fenestration and dehiscence may be challenging and may require cone-beam computed tomography (CBCT) for accurate diagnosis but the diagnosis of mucosal fenestration is relatively easy because of the clinical appearance and the shape of the defect, which is usually round with visible apical portions of the root [8]. Sensitivity and/or pain is rarely present in the affected tooth although it may sometimes occur on mastication or palpation [2]. The current case report describes the conservative treatment modality undertaken in a four-year-old child who presented with mucosal fenestration in the maxillary anterior labial gingivae.

## Case presentation

**Figure 1:**
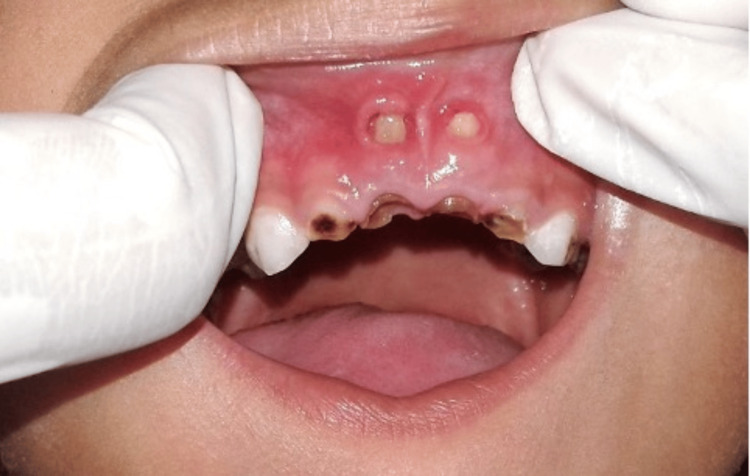
Fenestration exposing the root apices of grossly carious primary maxillary central incisors

A four-year-old child was reported to the Department of Pedodontics and Preventive Dentistry with the chief complaint of injury to the inside of the upper lip due to some hard projections on the upper gum. The patient had no previous relevant medical and dental history. Extraoral examination revealed no abnormalities. Intraoral examination revealed the presence of multiple grossly carious deciduous teeth with root stumps of the maxillary primary incisors. Mucosal fenestration (3 mm × 3 mm) exposing the root apices of the primary maxillary central incisors (teeth no. 51 and 61 as per the Fédération Dentaire Internationale (FDI) System)) was observed (Figure 1).

**Figure 2:**
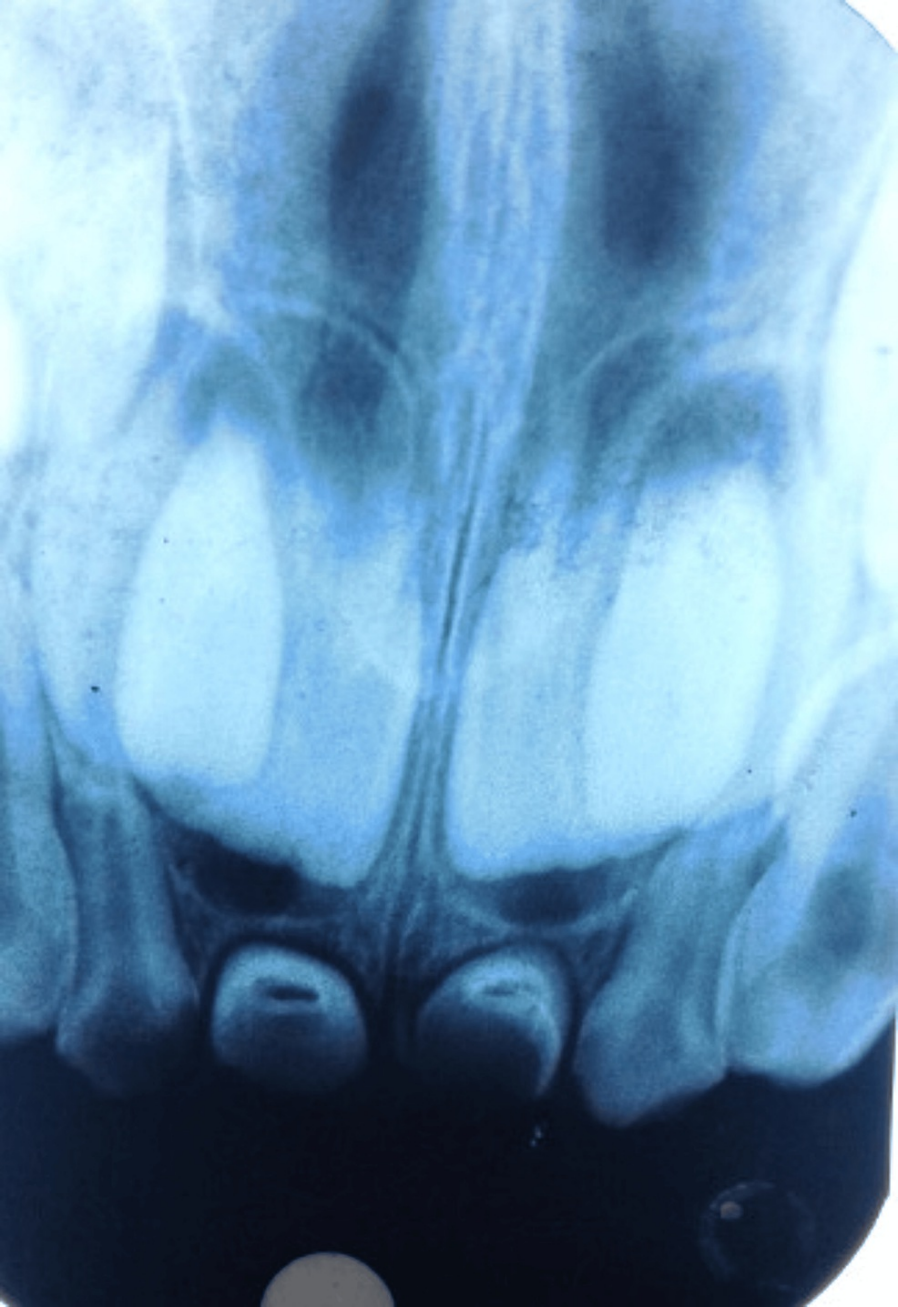
Intraoral periapical radiograph showing the absence of roots of the primary maxillary central incisors

The intraoral periapical radiograph revealed only the presence of crowns of the primary upper central incisors (Figure 2).

**Figure 3:**
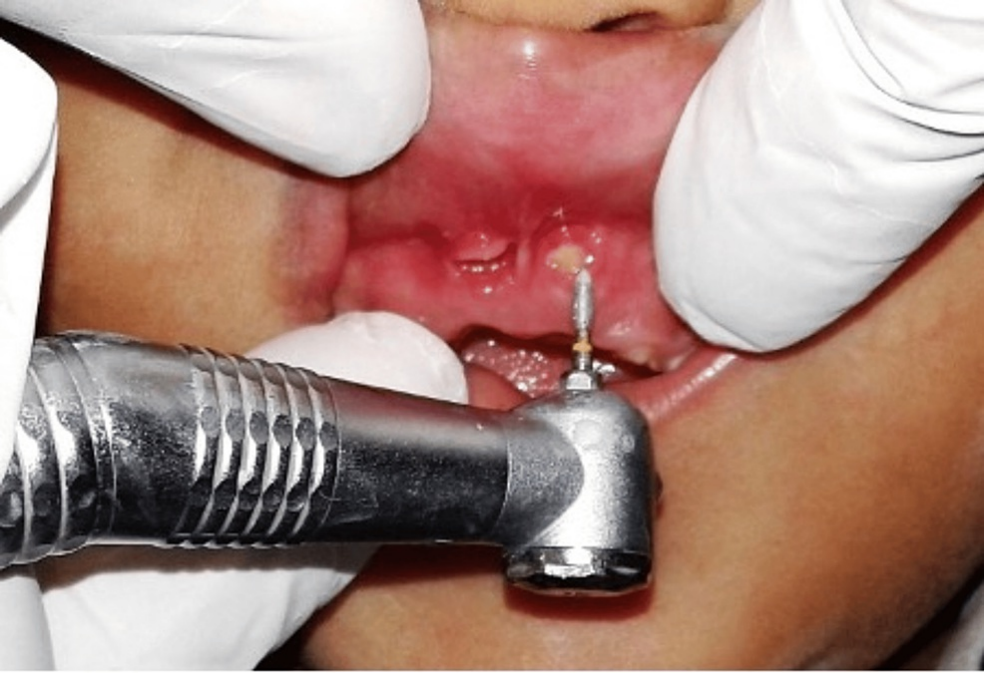
Grinding and resecting the root apices

Initially, the extraction of the central incisors was advised but the parents were against it. Also, the child’s behavior was negative (Rating No. 2 Frankl behavior rating scale). Hence, a conservative treatment plan was devised. The outline of the treatment is as follows. A topical anesthetic gel (Benzocaine 20%, ICPA Health Products Ltd., Mumbai, India) was applied on the mucosa surrounding the exposed root apices of the maxillary primary central incisors followed by labial infiltration anesthesia with 2% lignocaine (Xylocaine 2%, German Remedies, Zydus Health Care Ltd., Ahmedabad, India). A flame-shaped diamond-coated bur was inserted in a high-speed airotor handpiece. The bur was oriented parallel to the longitudinal axis of the root apices and was used to grind the apices completely (Figure 3).

**Figure 4:**
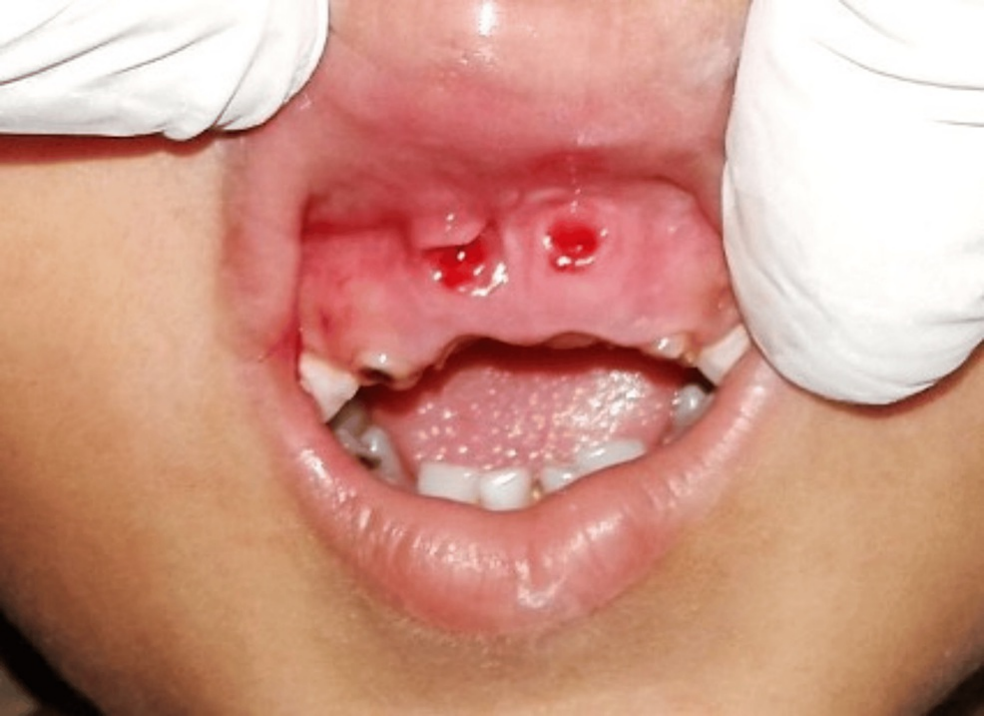
Immediate postoperative photograph

All the visible granulation tissues were enucleated. A pressure pack was placed and primary hemostasis was achieved (Figure 4). Analgesics and antibiotics were prescribed.

**Figure 5:**
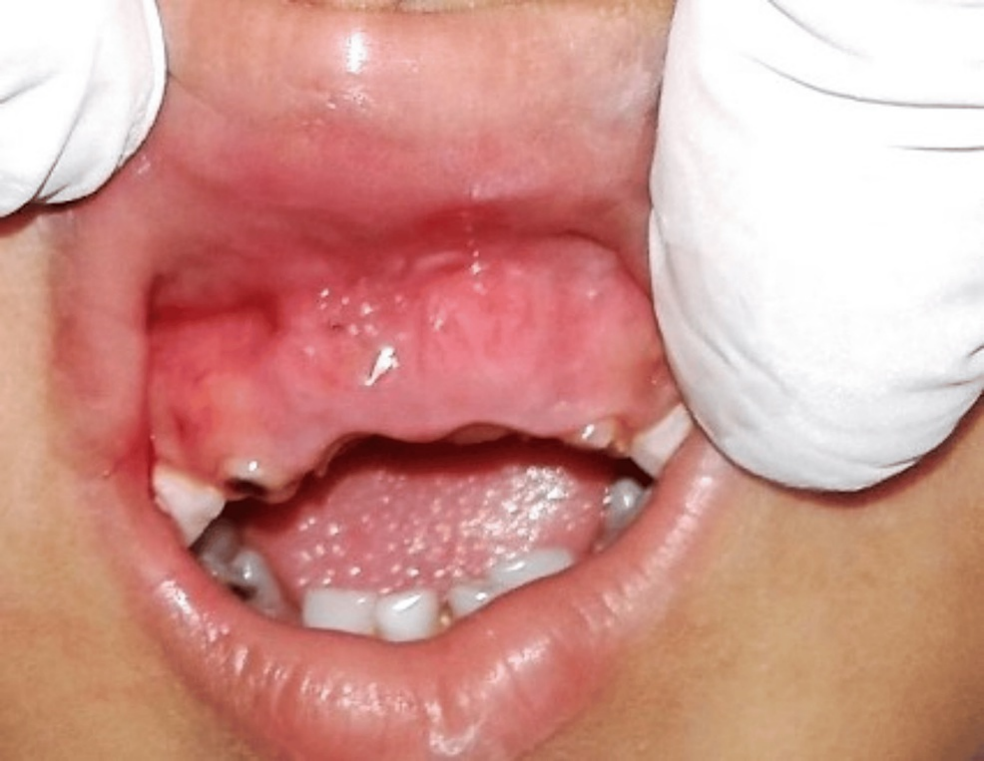
Follow-up after one year showing complete healing of the areas

At the end of a one-year follow-up, the previously fenestrated areas showed complete healing and resolution (Figure 5).

## Discussion

Fenestration of a root apex, resulting from pulpal and peri-radicular diseases is a rare phenomenon [5-7,9]. The foremost step toward devising a treatment plan for mucosal fenestration is to identify the underlying etiology [2].In primary teeth, fenestration can be a cause of trauma to the deciduous tooth in which its apex is found to perforate the labial alveolar plate and overlying gingival tissue. Another cause can be the prolonged retention of primary teeth beyond their exfoliation time. This is because the eruption of the permanent successor may cause resorption of the labial bone leading to fenestration, thus exposing the apex of the primary tooth [10]. The treatment usually undertaken for fenestration in primary teeth is extraction. For permanent teeth, alternative management methods comprise root canal treatment followed by root-end resection and retrograde filling with suitable restorative materials. Other than that, raising a full thickness flap followed by guided tissue regeneration and bone grafting can be done. Pedicle flap operations are another alternative. Thorough root planning should also be part of these surgeries [2]. The degree of anatomical misalignment and the extent to which the root apex protrudes beyond the alveolar bone are key factors in determining the necessary extent of root resection. However, it is possible to promote the closure of the mucosal defect by debriding the exposed root surface in combination with appropriate oral hygiene practices [8]. Regular maintenance of oral hygiene by daily brushing and flossing and, if necessary, rinsing with chlorhexidine mouth rinses are essential for the success of flap surgeries. Cases of mucosal fenestration at an early age of four years have rarely been documented in literature. Also, conservative management methods regarding such clinical presentations are very scarce to be found in scientific dental articles. In uncooperative pediatric patients, like in this scenario, simple grinding and resection of exposed root apices can serve as an efficient non-invasive management technique to relieve the fenestrated areas. Subsequently, any traumatic lesions resulting from such fenestrated defects can be managed in an effectually competent manner.

## Conclusions

Non-invasive dental procedures are best suited in the field of pediatric dentistry. Soft tissue trauma resulting from sharp exposed root apices of primary teeth due to fenestration can be alleviated through resection of the apices by diamond burs in high-speed handpieces without raising any surgical flaps. Thus, in this way, it not only produces good clinical outcomes but also creates a sense of satisfaction in the minds of the clinician and the parents.
